# Bioscreening and expression of a camel anti-CTGF VHH nanobody and its renaturation by a novel dialysis–dilution method

**DOI:** 10.1186/s13568-016-0249-1

**Published:** 2016-09-13

**Authors:** Xiulei Xue, Xiaobo Fan, Qingrong Qu, Guoqiu Wu

**Affiliations:** 1 Clinical Biochemistry Department, Medicine School, Southeast University, Nanjing, China; 2 Center of Clinical Laboratory Medicine of Zhongda Hospital, Southeast University, Nanjing, China

**Keywords:** Protein renaturation, Nanobody, Phage display, Connective tissue growth factor

## Abstract

The variable regions of the camel heavy chain antibody, also known as nanobody is the smallest antibody with antigen-binding efficiency. CTGF is considered important during extracellular matrix deposition which was involved in the pathogenesis of fibrosis related diseases. There are several anti-CTGF-C nanobody drugs under developing in pharmacy. In this study, we described the screening of a novel anti-CTGF-C nanobody from the peripheral blood of immunized camel by phage display. The screened nanobody was further expressed and purified from *E. coli* cells. A sophisticated dialysis–dilution method was designed for the in vitro refolding of the nanobody. The results showed that the expressed nanobody was consisted of 135 amino acid and mainly expressed as inclusion body in *E. coli* cells. The dialysis–dilution method was very effective and the recovery rate of the renaturation was more than 80 %. The ELISA result suggested the nanobody had been well refolded showing a superior CTGF binding activity to the commercial mouse anti-CTGF-C mAb. In conclusion, the anti-CTGF-C nonobody had been successfully screened by phage display. The dialysis–dilution refolding method was very effective and the recovery rate reached over 80 %.

**Electronic supplementary material:**

The online version of this article (doi:10.1186/s13568-016-0249-1) contains supplementary material, which is available to authorized users.

## Introduction

Connective tissue growth factor, also known as CCN2 is the second member of the CCN family which were characterized of four discrete modules, namely insulin-like growth factor binding protein-like (IGFBP), von Willebrand factor type C repeat (VWC), thrombospondin type 1 repeat (TSP1) and C-terminal cystine-knot (CT) modules (Leask and Abraham 2006; Kubota and Takigawa 2007). The full-length CCN2 is a 38-kDa protein with a tetramodular structure. These modules are commonly characterized of cysteine–rich domains and are highly interactive with numerous cellular factors, such as such as fibronectin and heparin sulfate proteoglycans, extracellular signaling molecules, and cell surface proteins (Holbourn et al. 2008). CTGF expression is spatio-temporally restricted and is involved in multiple physiological events, such as postnatal olfactory development, epithelial–mesenchymal transition (EMT) (Mosher and Adams 2012; Aguiar et al. 2014; Kubota and Takigawa 2015) and islets formation in the pancreas. In spite of its physiological contribution, CTGF is most widely recognized as a profibrotic factor. In most fibrotic disorders, CTGF acts as a downstream effector of TGF-β to promote the phenotypic conversion of fibroblastic cells to the myofibroblasts that conduct fibrosis (Ihn 2002; Dendooven et al. 2011). Besides, CTGF disorder is found in a variety of malignancy. CTGF is produced by tumor cells and acts on themselves through a paracrine manner promoting the tumor invasion and metastasis (Chu et al. 2008; Aguiar et al. 2014).

The camel heavy chain antibody consisted of only one heavy chain including a variable domain and two constant regions of CH2 and CH3 was first reported by Hamers–Casterman and his colleagues in 1993 (Hamers–Casterman et al. 1993). The variable domain of the camel heavy chain (VHH) also known as nanobody is the smallest antibody with antigen binding efficiency and is considered valuable in clinic due to its high tissue penetration efficiency (Kong et al. 2014). From a biotechnology view, nanobody is an ideal subject and is considered superior to the conventional antibodies such as IgG and scFv. Nanobody is easy for heterogeneous expression in eukaryotic or prokaryotic systems (van der Vaart 2002; Mizukami et al. 2015). Nanobody possessing multiple disulfide bonds is more stable and it could be stored in RT. Unlike scFv which always self-aggregates, nanobody is more soluble after heterogeneous expression (Kong et al. 2014).

The C-terminal module of CTGF is reported of potential to bind integrin receptors and to promote cellular adhesion and migration independently (Gao and Brigstock 2004; Hoshijima et al. 2006). In this study, we aimed to screen the VHH gene from the peripheral blood of a camel immunized with CTGF-C proteins by phage display, and then to express the VHH nanobody in *E. coli* cells as inclusion body. The expressed nanobody required renaturation and the in vitro activity was further investigated by ELISA after purification.

## Materials and methods

### Strain, Cell and DNA

*Escherichia coli* BLR (DE3) and BL21 (DE3) (New England Biolabs, Beverly, MA, USA) served as expression hosts. The pET22b(+)-anti-CTGF-VHH plasmid was used as the expressing vectors (GeneBank no. KX428017). The DNA primers were ordered from Generay Co., Ltd (Shanghai, China).

### Reagents

The CTGF-C protein was provided by ZhongDa Hospital (Nanjing, China). The CTGF/CCN2 standard sample was purchased from Peprotech (London, United Kingdom). LuriaBertani (LB) medium (w/v) was used for *E. coli* culture. Restriction enzymes *Nco*I and *Not*I were purchased from New England Biolabs. Mouse anti-CTGF-C monoclonal antibody was purchased from R&D (USA). Horseradish peroxidase-conjugated mouse anti-human CTGF polyclonal antibody were purchased from Abnova (USA). Nickle affinity column and Sephadex G-50 were purchased from Novagen (Madison, Wisconsin, USA). Other chemicals used in this study were of analytical or higher grade.

### Camel immune

A healthy 12-month-old male camel was first immunized with 200 mg recombinant CTGF through muscular injection, and the second and third injections were taken at 1 and 6 months later, respectively. Peripheral blood was collected at the 7th month from the jugular vein.

### RNA extraction and construction of camel VHH Antibody phage pool

A volume of 100 ml peripheral blood was centrifuged at 1000 rpm and the blood cells were collected. Red cells were removed by washing the cells in red cell lysate buffer for 4 times and the remains were collected for RNA extraction (Tanha et al. 2002; Miyazaki et al. 2015).

**Fig. 1:**
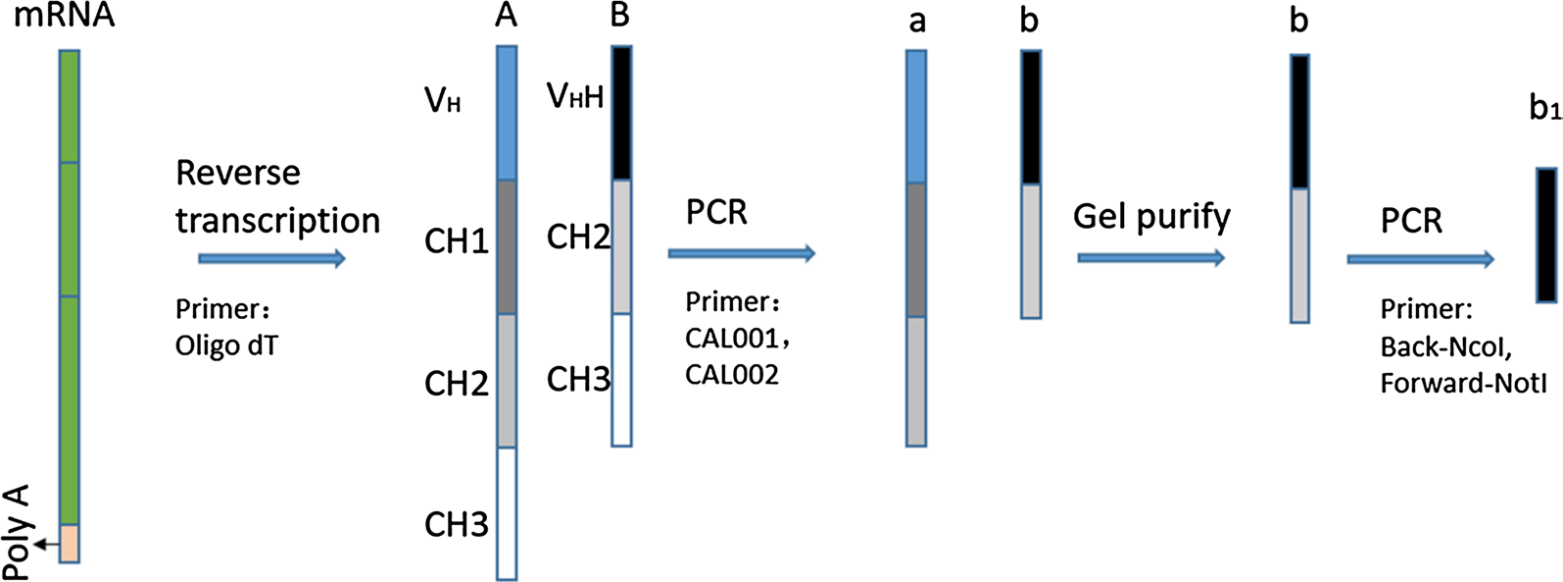
Schematics presentation of VHH library construction. cDNAs of *A* conventional antibody, *B* heavy chain antibody and their RT-PCR products of *a* and *b*. The VHH gene (*b1*) was obtained by another round of PCR

The construction of VHH antibody library of CTGF was schematically represented in Fig. 1. The total RNA was extracted from 3 × 10^7^ leukocytes by the QIAGEN RNA Blood kit (QIAGEN, Mississauga, Canada) according to the recommended protocol. The VHH repertoire was retrieved with reverse transcription followed by two rounds of nested PCR (Tanha et al. 2002; Fagerlund et al. 2014). In brief, RNA-derived cDNA was obtained by reverse transcription with an oligo-dT primer and 10 μg RNA as the template. The obtained cDNAs were subjected to another two rounds of PCR for amplification. There were two different forms of cDNAs, of which one contained only VHH and CH2 regions and the other contained an extra CH1 region. The primers for each PCR were summarized in Table 1. The PCR product from each round was purified from agarose gel. The VHH DNA repertoire was analyzed by sequencing.

**Table 1:** The primers used for the VHH library construction

Primer	Sequence
CALL001	5′-GTCCTGGCTGCTCTTCTACAAGG-3′
CALL002	5′-GGTACGTGCTGTTGAACTGTTCC-3′
Back-*Nco*I	5′-CCAAATCCATGGCCGAGTCTGGRGGAGG-3′
Forward-NotI	5′-CCAAATGCGGCCGCTGAGGAGACGGTGACCTGGGT-3′

About 4.1 μg purified VHH PCR fragments and 16.6 μg of linearized pHEN2 vector were used for ligation. VHH DNAs were digested with *Not*I and *Nco*I and then inserted into phage M13 vector which was pre-digested with *Not*I and *Nco*I. The constructed recombinant vector was applied to ~10^15^*E. coli* cells for transformation with electroporing, and the *E. coli* cells were then cultured in LB medium for 24 h at 37 °C. Phage particles were precipitated by addition of sterile 5 % PEG2000, and then resuspended in sterile PBS for bio-banning.

### Bio-panning of phage

A 96-wells plate was coated overnight at 4 °C with 20 μg/ml human CTGF C-terminal domain (CTGF/C, PeproTech, Asia) dissolved in 0.05 M NaHCO3 (pH8.6) (Nunc) (100 μl/well) followed an incubation with 5 % BSA. The phage was diluted to 3 × l0^12^ pfu/ml with TBS containing 0.1 % Tween-20 and 100 μl of the dilution was then added to each well of the plate with slightly shaking at an interval of 5 min at 37 °C for 1 h. The wells were washed with 0.1–0.5 % Tween-20/TBS to remove the unbound phages. The bound phages were retrieved by adding 0.2 M Glycine–HCl (pH2.2) containing 0.5 % BSA, and immediately neutralized with 1 M Tris–HCl (pH9.1).

The eluted phage with affinity to CTGF were added into exponentially growing *E. coli* BLR cells in 10 ml LB medium and stood for 30 min at 37 °C. Forty millilitre LB containing 100 mg/ml of ampicillin and 1 % of glucose was added and the flask was incubated at 37 °C for 2 h. Wild-type phage VCSM13 (Invitrogen Corp.) of 1 × 10^12^ pfu was added and incubated for another 4.5 h at 37 °C. The phage was collected from the supernatant after centrifugation at 8000 rpm for 15 min at 4 °C. The phages were further purified for the next round of bio-panning by precipitation with 1/6 volume of polyethylene glycol 8000 (PEG8000)/2.5 M NaCl. Three rounds of bio-panning were applied with the selection strength increased in each round as shown in Table 2.

**Table 2:** The panning conditions for each round

Panning rounds	Each well			Rinse times
	Input phage (pfu/ml)	Tween-20 (%)	CTGF-C (μg)	
1st	3 × l0^11^	0.1	2	5
2nd	4 × 10^10^	0.5	2	10
3rd	2 × 10^8^	0.5	2	15

### Phage ELISA and DNA sequencing

A small aliquot of the the purified phages after bio-panning was serially diluted in exponentially growing BLR cells to determine the titer. Two hundred microliters of the diluted cells were mixed with 3 ml of 0.7 % agarose in LB/IPTG/X-gal at 45 °C and immediately poured onto three plates prewarmed to 37 °C. Plates were incubated overnight at 37 °C and plaques were counted.

Individual plaque was randomly picked out, amplified and subjected to phage enzyme-linked immunosorbent assay (ELISA). In brief, the eluted phage with affinity to CTGF were added into exponentially growing BLR cells in 1 ml LB medium and stood for 30 min at 37 °C. Four millilitre LB containing 100 mg/ml of ampicillin and 1 % of glucose was added and the flask was incubated at 37 °C for 2 h. Wild-type phage VCSM13 (Invitrogen Corp.) of 1 × 10^11^ pfu was added and incubated for another 4.5 h at 37 °C. The phage was collected from the supernatant after centrifugation at 8000 rpm for 15 min at 4 °C. The phage was further purified by precipitation with 1/6 volume of polyethylene glycol 8000 (PEG8000)/2.5 M NaCl, and resuspended in 0.5 ml of PBS (pH7.4). An aliquot of 50 μl resuspension was added to each well of a microtiter plate that was pre-coated with 1 μg/ml of CTGF dissolved in 0.05 M NaHCO_3_ (pH 8.6) and blocked by 1 % BSA. After incubated at room temperature for 2 h, the plate was washed 15 times with 2.0 % Tween-20/TBS. The bound phages were detected by adding 50 μl HRP-conjugated anti-M13 antibody (Pharmacia, diluted 5000 times with PBS) followed by an incubation of 1 h. A volume of 50 μl 3,3′,5,5′-tetramethylbenzidine (TMB, Sangon, China) of 100 μg/ml was added to each well and incubated for 10 min. H_2_SO_4_ (1 M, 50 μl/well) was added and the plate was sent for colorimetry to measure the absorbance at 450 and 620 nm (Alisei, Italy). Fifteen colonies with the most absorbance at 450 nm were sent for sequencing.

### Expression and purification of VHH nanobody

The phage DNAs containing genes of the anti-CTGF/CCN2 VHH camel antibody were amplified by PCR with two VHH-specific primers of Back-NcoI and forward-NotI. After purified from agarose gel, an approximate 400-bp fragment was obtained with flanked *Nco*I and *Not*I sites. The DNA was inserted into the pET22b vector for expression.

After transformed with pET22b-VHH-His, *E. coli* BL21 cells were grown in 1L LB medium containing 100 μg/ml ampicillin at 37 °C overnight. The culture was then added with isopropyl β-d-thiogalactopyranoside (IPTG) to a final concentration of 0.2 mM and incubated for another 12 h at 18 °C. Cells were collected after centrifugation and then sonicated in rinse buffer (20 mM tris–HCl, 1 % Tritoon-X100, 1 mM DTT, 50 mM NaCl and 1 mM EDTA, pH 8.5) for 30 min at 300 W (1/1 s). The inclusion body were retrieved after centrifugation at 15,000×*g* for 30 min, and then resuspended in dissolution buffer (20 mM tris–HCl, 6 M Guanine-HCl, 1 mM DTT, 200 mM NaCl and 1 mM EDTA, pH 8.5) followed by stirring overnight at 4 °C. The dissolved supernatant was retrieved and then subjected to Ni–NTA chromatography according to the manufacturer’s manual. Finally, the target protein was eluted with elution buffer (2 mM Tris–HCl, 6 M Urea, 200 mM NaCl and 1 mM EDTA, pH 8.5). The protein samples during the expression and purification were analyzed with SDS-PAGE, and the concentration was determined by the Bradford method with a BCA kit.

### Renaturation of the nanobody by three methods

The elution containing 6 M urea was serial diluted with ddH_2_O and then centrifuged at 8000 rpm. The urea critical concentration (Ct) was defined as the lowest urea concentration where protein aggregation occurred. Before the experiment, Blue Dextran 10 or calcein was added into the upper chamber to estimate the diffusion rate of big or small molecules, respectively. The flow rate and the urea addition were adjusted accordingly to avoid precipitation of unfolded protein. The next step of protein renaturation was carried out at 4 °C unless stated otherwise.

### Dialysis–dilution method

**Fig. 2:**
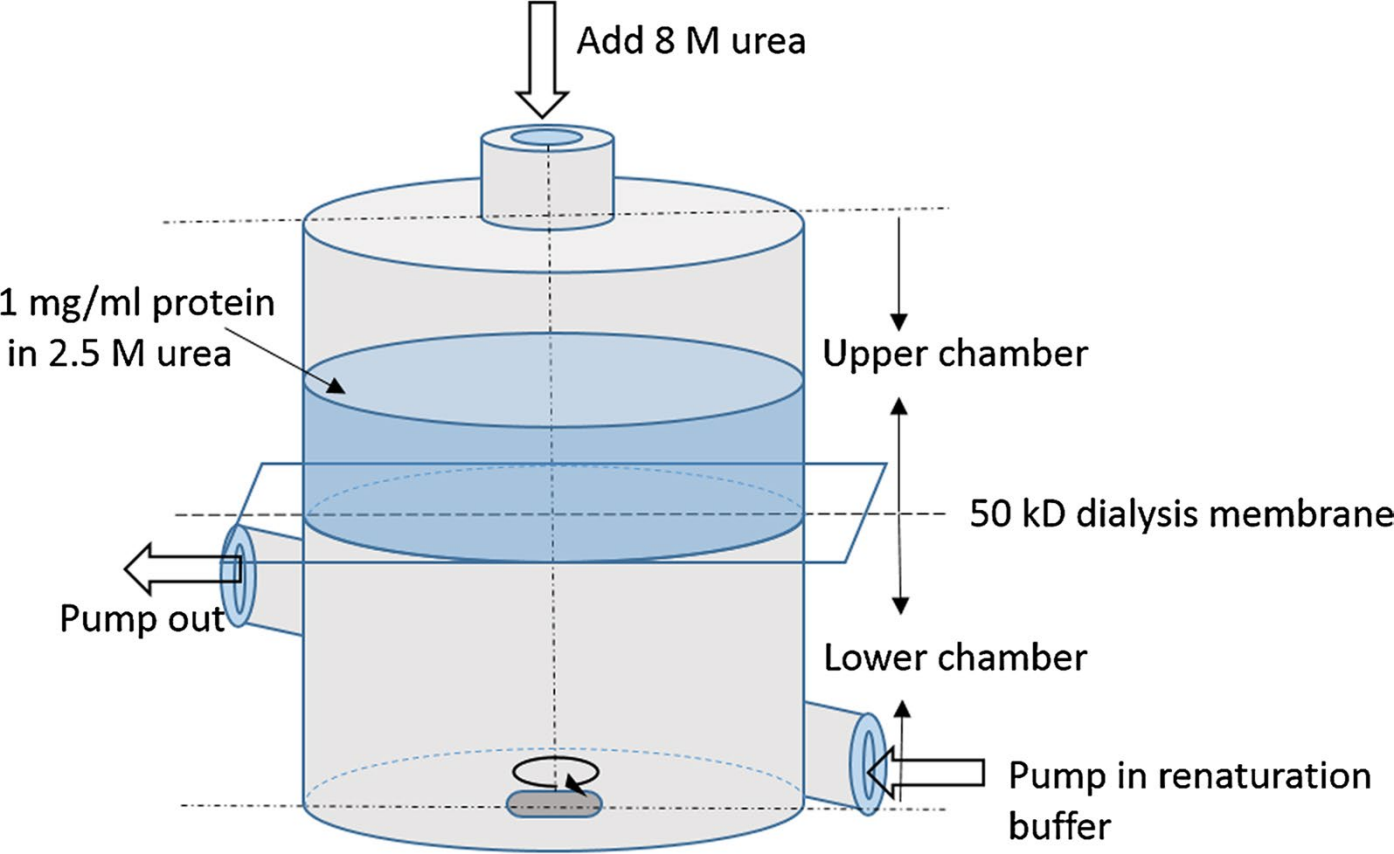
The schematics of the dialysis–dilution cassette

The elution after Ni–NTA chromatography was diluted to containing 2.5 M urea and 1 or 0.1 mg/ml protein for the next renaturation. The renaturation was performed with a modified dialysis–dilution method using settings as shown in Fig. 2. A volume of 10 ml dilution containing 0.1 or 1 mg/ml protein was added into the upper chamber while the lower chamber equipped with a magnetic rotator was filled with renaturation buffer (1 M urea, 20 mM Tris–HCl, 0.5 mM PMSF, 0.4 M arginine, pH 7.4) and the between was placed with a 50 kD dialysis cellulose membrane. Renaturation buffer was continually pumped into the lower chamber at 2 ml/min. The upper chamber was added with 0.8 ml 8 M urea every 10 min to keep the urea concentration above the urea Ct concentration and Blue Dextran 10 (MW 10,000 kD) was added in the upper chamber as indicator. The renaturation buffer through the chamber was collected, stirred overnight and then subjected to another round of Ni–NTA chromatography as described above followed by desalting with ultra-filtration (6 kD filter). The insoluble was removed by centrifugation and the final protein was lyophilized and stored in −20 °C for usage.

### Normal dialysis method

The elution was diluted with 2 M urea to a final concentration of 0.1 mg/ml protein. A volume of 10 ml dilution was stepwise dialyzed against 1.8, 1.6 or 1 M urea, and each concentration of urea was applied for 8 h. Aggregates were removed by centrifugation and the soluble protein was finally desalted with ultra-filtration.

### Normal dilution method

A baker equipped with a magnetic rotator was filled with 1 l renaturation buffer (1 M urea, 20 mM Tris–HCl, 0.5 mM PMSF, 0.4 M arginine, pH 7.4). The elution was diluted with 2 M urea to a final concentration of 0.1 mg/ml protein. A volume of 10 ml dilution was continuously pumped into the renaturation buffer at a constant rate of 0.1 ml/min. The soluble protein was retrieved and finally desalted with ultra-filtration.

### Circular dichroism spectrum

The elution after Ni–NTA chromatography was diluted with 2 M urea to a final concentration of 1 mg/ml protein while the protein samples after renaturation were dissolved in ddH_2_O at 1 mg/ml. The prepared samples were measured with a Jasco-810 spectropolarimeter (JASCO, Tokyo, Japan) using a 1-mm path-length quartz cell at 25 °C. Each data was collected as the average of four scans in the wavelength between 190 and 250 nm, with a 1 nm step resolution at 100 nm/min speeds. For a flexible peptide, the estimated percentages of secondary structure components should not be taken as absolute measures, but rather reflect on relative changes between spectra in a series of experiments.

### Immunoreactivity assay by ELISA

The affinity of the purified VHH antibody with CTGF was evaluated by ELISA. The VHH or mouse anti-CTGF-C mAb was immobilized onto a 96-well microtiter plate by adding 200 μg/ml × 100 μl protein to each well followed by an overnight incubation at 4 °C. The uncoupled protein was washed away and 100 μl 1 % BSA was added to block the uncoupled sites. The plate was incubated at 37 °C for 2 h after 50 μl of CTGF ranging from 256 to 0.125 μg/ml was added to each well. Afterwards, a volume of 50 μl s antibody (1:4000 dilution, horseradish peroxidase-conjugated mouse anti-human CTGF polyclonal antibody) was added. The plate was washed with 2.0 % Tween-20/TBS before each round of addition. A volume of 50 μl 3,3′,5,5′-tetramethylbenzidine (TMB, Sangon, China) of 100 μg/ml was added to each well and incubated for 10 min. H_2_SO_4_ (1 M, 50 μl/well) was added and the plate was sent for colorimetry to measure the absorbance at 450 nm (Alisei, Italy).

## Results

### Construction of immunized VHH library

**Fig. 3:**
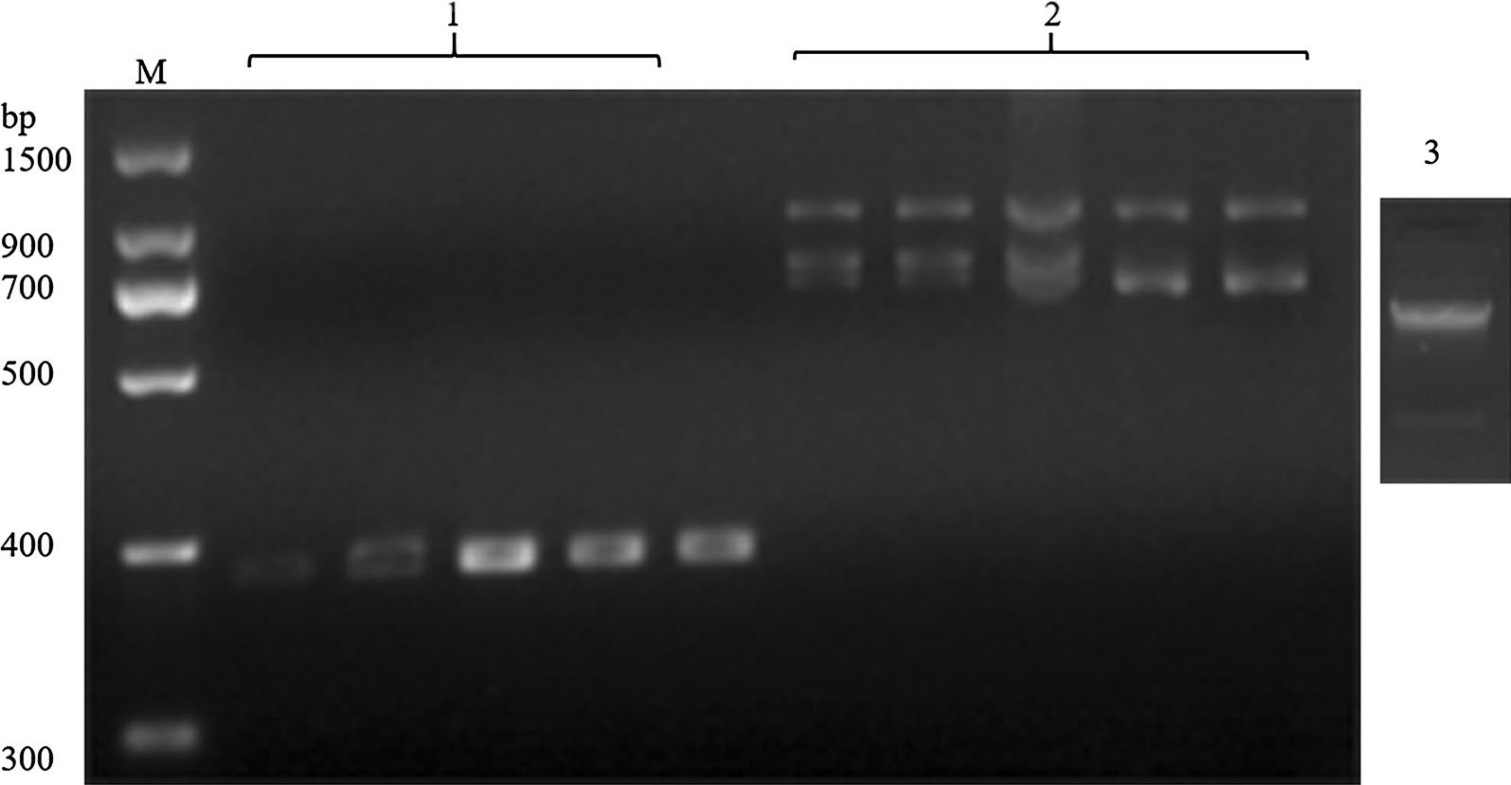
The retrieve of VHH mRNA and the DNA after each round of nested PCR. Lane M DNA marker; *lane 1* the PCR products in the second nested PCR; *lane 2* the PCR products in the first nest PCR; *lane 3* the extracted mRNAs from the leukocytes

For construction of camel VHH library, a healthy Bactrian camel was immunized with purified CTGF-C over a period of 7 weeks. After the final immunization, the total IgG titer of immunized serum reached 1:12,800 indicating the success of raising immunogenic response in the camel. The RNA extracted from PBMC was analyzed by agarose gel and the result showed that there were major two binds (Fig. 3 lane 3). The antigen-binding gene fragments of the heavy-chain antibody variable region were amplified from lymphocyte cDNA by nest PCR. In the first round of PCR amplified with primers CALL001 and CALL002, the resulting PCR fragments had two distinct bands at around 700 and 900 bp corresponding to VH-CH1-CH2 and VHH-CH2 genes, respectively (Fig. 3 lane 2). In the second nested PCR, only the VHH gene was retrieved with a size of ~400 bp (Fig. 3 lane 1).

According to the alignment (NCBI-blastx), the VHH DNA consisted of six regions: FR1 (1–60 bp), CDR1 (61–90 bp), FR2 (91–138 bp), CDR2 (139–162 bp), FR3 (163–201 bp) and CDR3 (202–303 bp). The VHH genes differed in 105 loci from the three CDR regions and the calculated repertoire reached over 10^20^.

### Phage panning

**Fig. 4:**
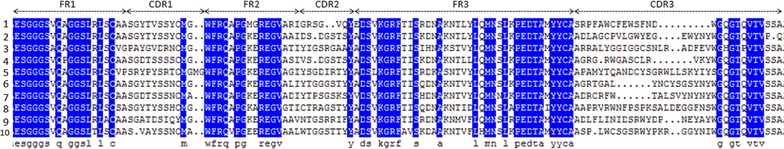
The alignment of the deduced protein sequences of 15 phage colonies after three rounds of bio-panning. The first sequence from the alignment was confirmed by three sequencing results. The second and the third were confirmed by two sequencing results

The VHH repertoire was expressed on phages after rescuing with VCSM13 helper phages. The results of the phage ELISA visualised a strong enrichment of antigen-specific clones during the consecutive rounds of panning (Table 3). The number of phage particles eluted from wells after each round was shown in Table 3 as well. Following three rounds of panning against CTGF, 15 colonies were randomly chosen for sequencing. The sequencing results indicated there were ten different sequences and their amino acid sequences were shown in Fig. 4. Analyzed by alignment, the VHH consisted of five regions. The three frame regions were conserved while the CDRs differed greatly between each other. The tenth sequence confirmed by three sequencing results was used for protein expression.

**Table 3:** The enrichment of the phage in during the bio-panning

library	Input phage (pfu)	Output (pfu)	Gene polymorphism loci in^a^			Calculated library size^b^
			CDR1	CDR2	CDR3	
VHH library	NA	NA	7	12	86	NA
1st panning	3 × 10^10^	1.36 × 10^5^	3	8	15	6.71 × 10^7^
2nd panning	4 × 10^9^	3.19 × 10^6^	NA	NA	NA	NA
3rd panning	2 × 10^7^	1.92 × 10^6^	4	4	3	3072

*NA* undetected or unmeasured

^a^Loci where gene sequencing results indicating presence of different nucleotides

^b^The calculated size should be much greater than actually size due to gene coupling

### Expression and purification of the nanobody

**Fig. 5:**
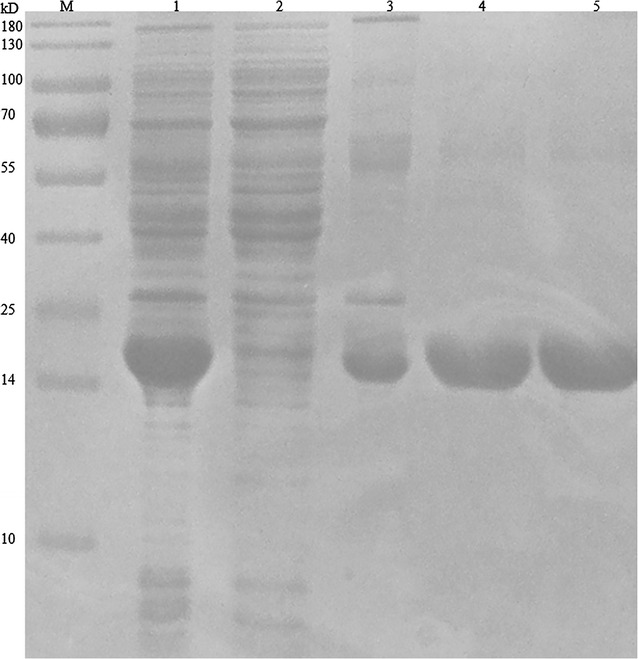
The expression and purification of the VHH nanobody from *E. coli*. Lane M marker; *lane 1* precipitates after cell lysis; *lane 2* supernatant after cell lysis; *lane 3* elution of Ni–NTA chromatography; *lane 4* the VHH antibody renatured by the dialysis–dilution method; *lane 5* the VHH antibody desalted by ultra-filtration

According to the deduced amino acid sequences, the expressed antigen-specific VHH nanobody was consisted of 135 amino acids with a theoretic M.W. of 14,464.12, and the pI was 7.84. The nanobody fused with a His-tag was mainly expressed as inclusion body and was retrieved by high affinity Ni–NTA column by denaturation method according to the manufacture manual. Sodium dodecyl sulfate polyacrylamide gel electrophoresis (SDS-PAGE) analysis showed that more than 75 % purity nanobody was obtained (Fig. 5). Moreover, the purified VHH was yielded on milligram quantity of 5.4 mg per liter of culture.

### Renaturation of the nanobody by three different methods

#### Dialysis–dilution method

The elution after Ni–NTA chromatography containing 6 M urea was diluted and then stored in 4 °C overnight. We found that the proteins within the dilutions would precipitate at urea concentrations below 2 M. So the urea critical concentration where proteins start to aggregate was determined as 2 M. The elution was therefore diluted to containing 2.5 M urea for renaturation.

**Fig. 6:**
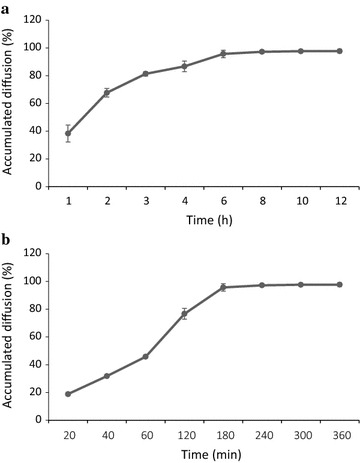
The diffusion rate of the dialysis–dilution setting. **a** The diffusion rate measured with Blue Dextran 10. A standard curve was made by preparing Blue Dextran 10 concentration serials and measuring with absorption at 620 nm. The Blue Dextran 10 was added into the *upper* chamber and dialyzed against the renaturation buffer. 100 μl samples were taken at different time points and measured with the absorption at 620 nm. The input flow rate at the *lower* chamber was 2 ml/min. **b** The diffusion rate measured with Calcein. The exiting and emitting wavelengths were 495 and 515 nm, respectively, and the diffusion rate was measured by a method similar to Blue Dextran 10

Before the renaturation, 20 ml × 1 mg/ml Blue Dextran 10 or 20 ml × 2.5 M calcein was added into the upper chamber to estimate the diffusion rate of big or small molecules, respectively. The results indicated >96 % blue dextran 10 could diffused across the 50 kD membrane within 8 h while calcein could reached 45.7 % within 1 h (Fig. 6). Therefore, the renaturation of the VHH antibody was performed for 8 h and 0.8 ml × 8 M urea was added into the upper chamber every 10 min to maintain the urea concentration from the upper chamber. No apparent precipitation had been observed during the dialysis when either 0.1 or 1 mg/ml startup protein samples were used. The protein after renaturation was well soluble in ddH_2_O and the size in SDS-PAGE gel was in consistent with before dialysis (Fig. 5). The recovery yield reached over 85 %.

#### Normal dilution and dialysis methods

The normal dilution method seemed very sufficient and yielded clear solution after dilution. However, the clear solution soon became cloudy after stored in 4 °C overnight and TEM results indicated that there was a lot of particles present in the dilution (Additional file 1: Figures S1, S2). After removing the particles, the final recovery rate was about 13 %.

Almost all proteins precipitated after normal dialysis and no further research was carried out to study the dialysis method.

### Circular dichroism spectrum

**Fig. 7:**
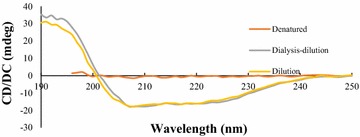
The secondary structure of the nanobody by circular dichroism. The protein sample dissolved in 2 M urea did not show any 2-D structures. The protein samples after renaturation by dialysis–dilution or normal dilution method showed similar 2-D structures were 7 % alpha-helix and 45 % beta-sheet

The results indicated the samples renatured by two different methods showed similar secondary structures that were 7 % alpha-helix and 45 % beta-sheet. The elution after chromatography dissolved in 2 M urea did not show apparent 2-D structures (Fig. 7).

### Affinity and sensitivity detection

**Fig. 8:**
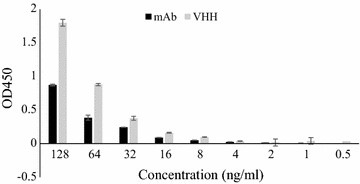
The CTGF-C binding affinity of VHH and mAb

The activity of the nanobody recognizing CTGF was performed by ELISA and the results displayed that the OD450 value increased together with the concentrations of the CTGF ranging from 0.125 to 128 μg/ml. In comparison with the commercial anti-CTGF-C mAb, the VHH antibody showed a superior CTGF-binding activity at the same weight concentrations (Fig. 8).

## Discussion

Connective tissue growth factor (CTGF/CCN2), a member of the CCN family, is a secreted protein with major roles in angiogenesis, chondrogenesis, osteogenesis, tissue repair, cancer and fibrosis (Kapoor et al. 2008; Leask 2008) Each module has specific binding affinity for certain proteins, such as fibronectin and heparin sulfate proteoglycans, extracellular signaling molecules, and cell surface proteins (Kubota and Takigawa 2015). The N-terminal domain (modules I and II) has been shown to mediate differentiation and collagen synthesis, and the C-terminal domain (modules III and IV) has been shown to regulate cell proliferation (Gao and Brigstock 2004; Hall-Glenn and Lyons 2011). A recently study showed that C-terminal domain of CTGF was associated with cell proliferation in leukaemia (Welch et al. 2015). It has been reported that the C-terminal domain of CTGF could stimulate cellular adhesion via the αv-β3 receptor (Ball et al. 2003; Hoshijima et al. 2006) and was involved in the fibrosis process (Rodrigues-Diez et al. 2015). Therefore, it is possible that the C-terminal domain of CTGF can be a potential novel pharmacological target of profibrotic disease such as using neutralizing antibodies, antisense oligonucleotide and inhibitors.

Unlike a mouse which was usually sacrificed for lymphocyte collection from the spleen, the camel was kept alive after this study and probably could be used in another experiment after immune homeostasis. After immunization with CTGF-C, the lymphocytes encoding the anti-CTGF-C antibodies circulated in the blood vessel. We collected the peripheral blood and retrieved the lymphocytes for mRNA extraction. After reverse transcription and two rounds of PCR, the VHH library has been successfully constructed. Unlike unimmunized library, the phage titration in the first round of panning reaching 10^6^ pfu/ml was much higher than previous reports implying the animal was well immunized and a lot of anti-CTGF-T-antibody expressed lymphocytes were present in the peripheral blood circulation (Tanha et al. 2002; Miyazaki et al. 2015). We applied two extra rounds of phage panning with extremely washing condition to screen the phenotype with the highest affinity to CTGF-C. The expressed VHH nanobody of 144 amino acids was much smaller than the mAb antibody which generally contained more than 500 residues. Therefore, as shown by the ELISA result, the VHH showed a superior CTGF-C-binding activity to mAb at the same mass concentration as there were much more VHH molecules than mAbs. The high affinity of the VHH nanobody could be attributed to two aspects: (1) low molecules weight resulting in less stereospecific blockade allowed more VHH molecules to target on one single CTGF-C protein; (2) extremely panning procedure resulted into the highest affinity of screened VHH nanobody.

The results showed the VHH antibody had been successfully expressed as inclusion body in *E. coli* cells, and the VHH antibody fused with a His tag could be easily purified from the cell lysate with a purity over 75 % by Ni–NTA chromatography. After renaturation, the VHH antibody was subjected to another round of Ni–NTA and the result showed the purity was further improved. A purity over 90 % was obtained in the final product.

**Fig. 9:**
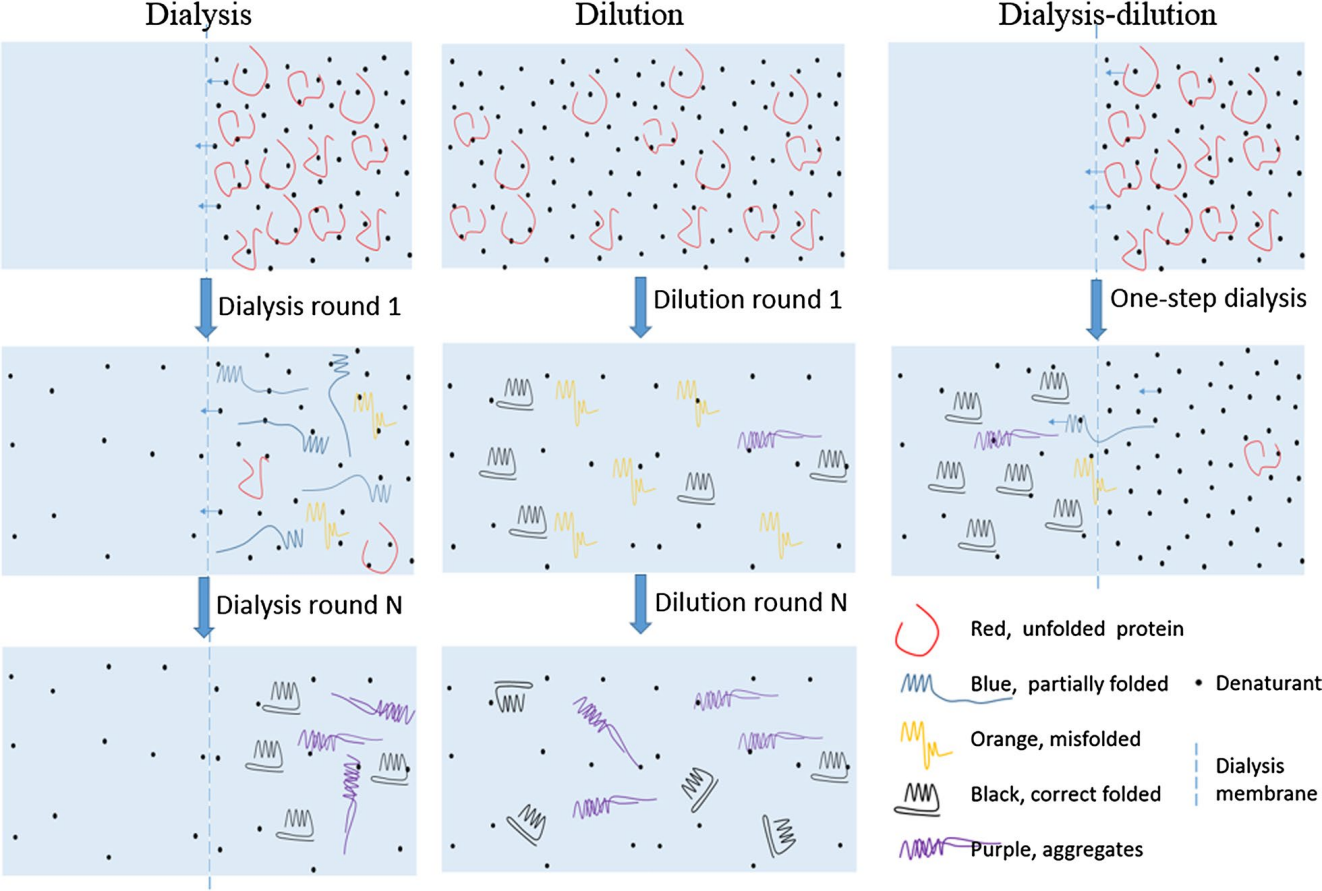
Schematics of the dialysis–dilution method in comparison with the two conventional methods. The conventional dialysis was a time-consuming process allowing the protein to fold slowly into the correct form. The conventional dilution method was a time-saving process but always yielded misfolded products. The dialysis–dilution method combined the merits of both

The inclusion body was renatured with a dialysis–dilution method. The dialysis cassette was specially designed for this method. The denatured VHH antibody was added into the upper chamber and then slowly diffused across the membrane which was totally different from the conventional dialysis renaturation as shown in Fig. 9. The conventional dialysis and dilution methods were ineffective in our case and gave a total recovery of <5 and ~13 %, respectively. The protein refolding was a time-consuming process and the dialysis method taken hours gave enough time for proteins to refold. During the dialysis method, the protein was retained in the dialysis bag and the proteins would start to refold while the denatured chemicals diffused outward. However, the dialysis was a complicated and time-consuming method requiring numerous repeats to optimize the refolding conditions and buffer composition, and generally taken multiple steps of dialysis (Jungbauer and Kaar 2007; Zilinskas and Sereikaite 2011; Berg et al. 2012). As we known, during the protein refolding, the major obstruction came from the self-aggregation (Zilinskas and Sereikaite 2011; Gautam et al. 2012). Therefore, dilution was an effective method for in vitro protein refolding as it allowed the protein to refold at a very low concentration thus to reduce the interference from self-aggregation (Gautam et al. 2012). Our results demonstrated that the dialysis method was less effective than the dilution method. However, the dilution method was difficult to manipulate because the protein molecules needed to be separated into the solution instantly that required special equipment. Self-aggregation was also reported as the major obstacle for the dilution method in many cases (Zilinskas and Sereikaite 2011; Gautam et al. 2012). To our surprising, the nanobody did not precipitate instantly after misfolded by normal dilution method but form small nanoparticles. The modified dialysis–dilution method combined the merits of both. Firstly, proteins diffusing across the membrane took hours allowed the protein to refold slowly into the correct form; secondly, the proteins diffused across the membrane as a single molecule therefore greatly reducing the self-aggregation. The CD results indicated the soluble proteins after different renaturation methods showed similar 2-D structures, suggesting that only well-folded nanobody was soluble while the unfolded and misfolded proteins aggregated.

The anti-CTGF-C VHH antibody had been successfully expressed and purified from *E. coli* cells. The ELISA results indicated the VHH nanobody after renaturation was bioactive and could specially bind CTGF with a superior affinity to mAb. The dialysis–dilution was a one-step, time-saving, economic and effective method, providing a promising alternative for industrial and laboratorial application of protein renaturation.

## Additional file

10.1186/s13568-016-0249-1 The aggregated particles after normal dilution by dynamic light scattering. The protein samples immediately after normal dilution were diluted 100 times with PBS before sent for dynamic light scattering measurement. The results indicated there were numerous particles of several hundreds of nanometer in diameter. **Figure S2.** The aggregates after normal dilution by TEM.

## Abbreviations

CTGF/CCN2: connective tissue growth factor
CTGF-C: connective tissue growth factor C-terminal
VHH/nanobody: variable domain of the camel heavy chain
IGFBP: insulin-like growth factor binding protein-lik
VWC: von Willebrand factor type C repeat
TSP1: thrombospondin type 1 repeat
CT: C-terminal cystine-knot
EMT: epithelial–mesenchymal transition
SDS-PAGE: sodium dodecyl sulfate polyacrylamide gel electrophoresis
