# Supplementary material for: Bioscreening and expression of a camel anti-CTGF VHH nanobody and its renaturation by a novel dialysis–dilution method
Source: AMB Express. 2016 Sep 13;6(1):72. doi: 10.1186/s13568-016-0249-1 (PMC5019992; doi:10.1186/s13568-016-0249-1)
Supplement: Supplementary file 1 — 10.1186/s13568-016-0249-1 The aggregated particles after normal dilution by dynamic light scattering. The protein samples immediately after normal dilution were diluted 100 times with PBS before sent for dynamic light scattering measurement. The results indicated there were numerous particles of several hundreds of nanometer in diameter. Figure S2. The aggregates after normal dilution by TEM. [file 13568_2016_249_MOESM1_ESM.docx]

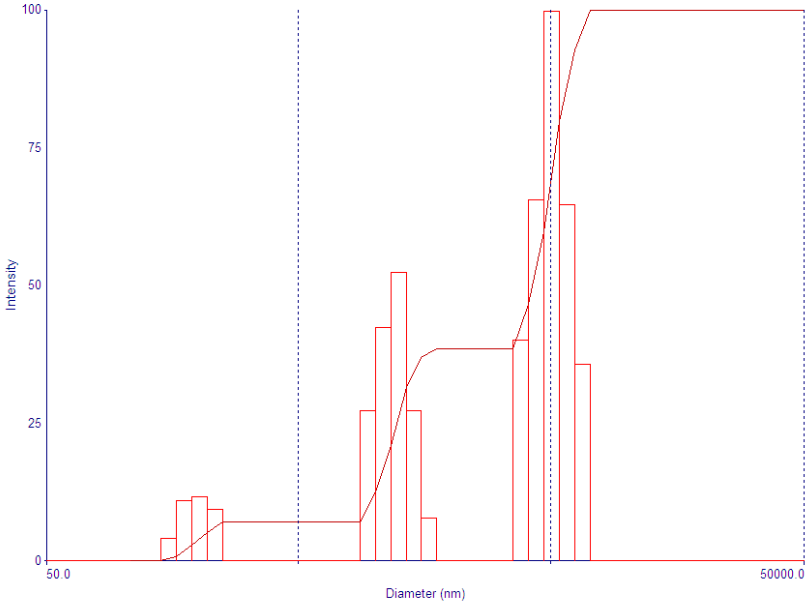


Figure S1. The aggregated particles after normal dilution by dynamic light scattering. The protein samples immediately after normal dilution were diluted 100 times with PBS before sent for dynamic light scattering measurement. The results indicated there were numerous particles of several hundreds of nanometer in diameter.





Figure S2. The aggregates after normal dilution by TEM.
